# New classification of branching pattern of facial nerve during parotidectomy: A cross sectional study

**DOI:** 10.1016/j.amsu.2021.01.006

**Published:** 2021-01-14

**Authors:** Omar Salem Khattab Alomar

**Affiliations:** Department of General Surgery, College of Medicine, University of Baghdad, P.O.Box: 19503, Zayona, Baghdad, Iraq

**Keywords:** Parotidectomy, Facial nerve, Facial nerve palsy, Classification of branching pattern

## Abstract

**Background:**

Parotidectomy is one of the most frequent modes to treate tumors of parotid gland. Previous studies documented a variation in the facial nerve branching which might risk facial nerve injury during Parotidectomy.

**Aim of study:**

To make a new classification system that includes a new branching pattern of facial nerve trunk that has not been described before, also to mention a simple method of how to identify the facial nerve trunk, all that will help the new surgeon in performing parotidectomy with less complications and unpredictable outcome.

**Methods:**

A prospective cross sectional study on 460 patients underwent partial or total parotidectomy for different pathologies were enrolled during the period January 2004 till September 2020. Three investigations were considered; the anatomy of the facial nerve trunk (FNT), exact site of facial nerve trunk in relation to fixed landmarks, finally we observed any communications between the branches. We made a new classification based mainly on the anatomical variations in the branching pattern of the FNT; namely, types (I, II and III). Each type subdivided according to the length of facial nerve trunk and also according to the communication between the branches.

**Results:**

Type I reported in majority of cases; 78.26%. type II (15.2%) which is the newly discovered branching pattern, and type III (6.6%). Total FNT length was 1–10 mm in more than half (54.35%) of cases. In 64.35% of cases FNT was in the midpoint between the tragal pointer (TP) and tip of mastoid's process (TMP). In 50 (10.87%) of the cases there was anastomotic connection between the buccal and mandibular branches, and in 20(4.34%) the communication was always a loop between the upper and lower divisions of FNT.

**Conclusion:**

There is a profound variation in the facial nerve branching pattern that has not been previously reported. Awareness about differences in the anatomy of the facial nerve assisted useful information to surgeon to preserve FN during parotidectomies.

## Highlights

• Previous studies documents variation in the facial nerve branching which might risk FN injury during parotidectomy.
• A new classification system is made that includes a new branching pattern of FN trunk that has not been described before.
• a simple method of how to identify the facial nerve trunk.
• All that will help the new surgeon in performing parotidectomy with less complications and unpredictable outcome.

## 1 Introduction

Parotidectomy is a practical surgical treatment for benign or malignant tumors of the parotid gland. However, weakness of facial nerve is the commonest complication in parotidectomies [[1], [2], [3]]. In majority of patients, FN weakness is transient, and full recovery usually occurs in up to 6 months post operatively [4,5]. Recent studies, indicated that 12 months-postoperative FN paralysis contributed for 9% of cases [2,[4], [5], [6]].

Identification of FN during parotidectomy is essential to evade its weakness or paralysis. Before any surgery on the parotid gland, good knowledge about anatomical variations and branching of FN is so important. When facial nerve protrudes from the stylomastoid foramen and permits throughout parotid gland giving its two divisions; the temporal and cervical, which are then subdivided to five terminal branches; Temporal, zygomatic, Buccal, mandibular and cervical [[4], [5], [6]].

Proper postoperative outcomes for surgical treatment of parotid tumors are mainly related to better exposure and conservation of facial nerve that needs good information regarding anatomy of facial nerve and high attention to any anastomosis or variation between the branches. For many previous centuries, the physicians stray the anatomy of parotid gland due to pathway of facial nerve and branching to temporofacial and cervicofacial with further terminal branches; temporal, zygomatic, buccal, marginal mandibular and cervical branches. The style of facial nerve divisions is not regular and this finding is confirmed by various sources [7,8].

In 1956, Davis et al. [7] were the first who described the FN branches and described 6 types of FN, I, II, III, IV, V and VI, depending on anastomosis existed among the terminal branches. Katz and Catalono, in 1987 adopted a new classification which did not match that of Davis [9]. It had 9 types of branches these are I-A, I–B, II, III-A, III-B, III-C, IV-A, IV-B and V. The origin of buccal nerve, the anastomosis between terminal branches and number of FN terminals represented the base for this classification. Later, in 1994, Kopuz et al. adopted an improved new version with three additional dual-trunk types including (VA, VB and VC) [10].

Most of the available classifications concentrate mainly on terminal branches of FN and the anastomosis between them, while during parotidectomy what is important for any surgeon is first to identify the trunk of facial nerve, then identifying all the terminal branches regardless the anastomoses between them. So we aimed to mention a simple method of how to identify the facial nerve trunk in relation to fixed landmarks, also we make a new classification based mainly on the anatomical variations of the FN trunk branching-patterns that we observed among Iraqi patients during parotidectomy including a new branching pattern of facial nerve trunk that has not been described before, and also described the terminal branches pattern, all these will help to provide a map for the operating surgeons, especially newly graduated young surgeons to decrease the injuries of FNs during parotidectomy and reduction of morbidities and postoperatively incident complications related to injuries of FN. The objective of the current study was to assess facial nerve branching variation in Iraqi population.

### 1.1 Patients and methods

A prospective cross sectional study conducted at the Medical City teaching hospital and Private Hospitals in Baghdad, during the period from 1st of January 2004 to 30th of September 2020. Inclusion criteria were Iraqi patients with parotid tumors who underwent partial or complete parotidectomy for various pathologies. Patient was excluded if there was a fixation of the tumor to the overlying skin, pre-operative FN palsy or recurrence of parotid tumor or refused to participate. The final sample of 460 patients with parotid tumors was selected after eligibility to inclusion and exclusion criteria.

Ethical considerations were obtained according to Helsinki Declaration. Informed written consent was obtained after explaining the nature of the operation and its risks. The approval of ethics committee was obtained from Health Ethics Committee in Baghdad Medical city teaching hospital. The patient was placed at a 15-degree angle to reduce venous congestion. The sandbag was placed under the shoulder on the same side and the head was moved away from the surgeon and the skin was also prepared. The methods of this article was prepared according to STROCSS criteria [11]. The research was regestered with numbers of 6255 at research registry [12].

A preauricular incision was made in the skin then, along with the inferior edge of mandible, the incision was extended along mastoid process, that what modified Blair's incision. Skin, subcutaneous tissue and superficial fascia were detached and retracted medially to masseter muscle edge to expose the parotid gland, which was separated from the external cartilaginous auditory canal until the entire cartilaginous canal was freed up.

**Fig. 1:**
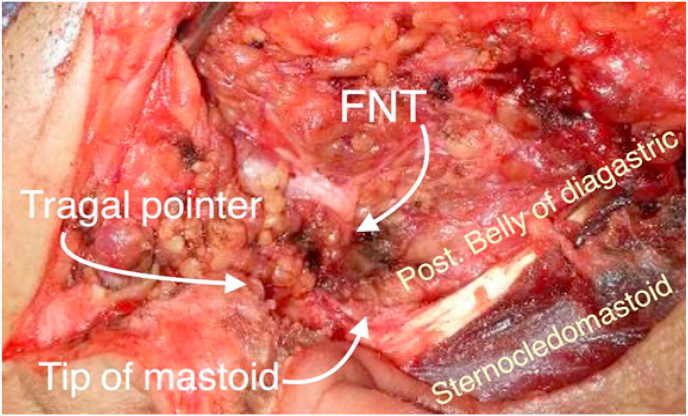
The landmarks used for facial nerve identification.

**Fig. 2:**
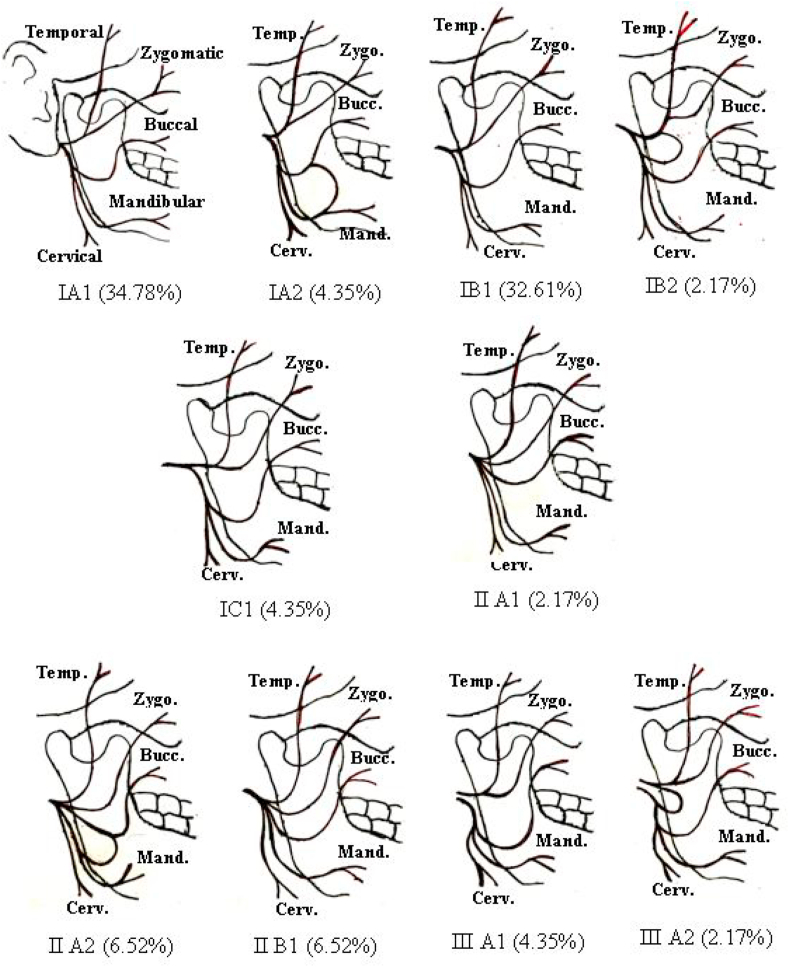
Facial nerve branching pattern types.

**Fig. 3:**
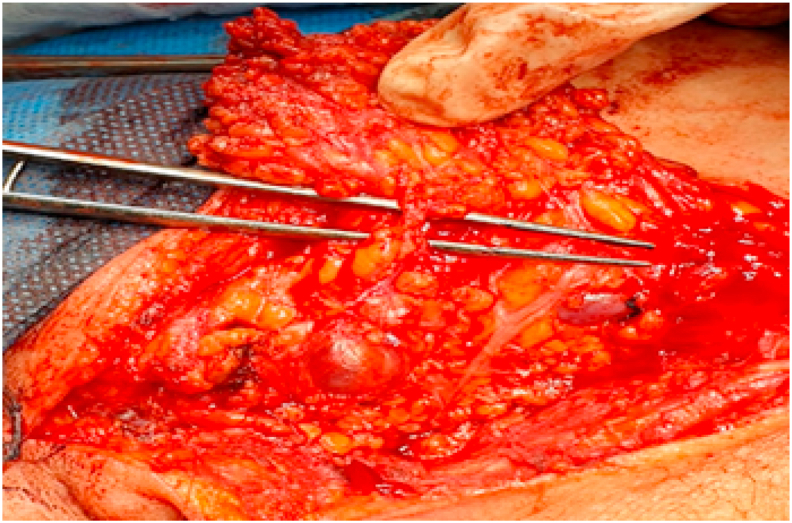
Identification of parotid duct.

In this study, tragal pointer (TP) and the tip of the mastoid processes (TMP) were used as landmarks to identify the facial nerve (Fig. 1). Dissection was firstly started in the middle point between these two landmarks. If the FNT was not indefinable, at this point, we first go 2 mm below toward TMP, if we do not specify there, we go 2 mm above the mid-point towards the TP. The nerve was localized by opening an arterial forceps parallel to the nerve until the FN trunk and terminal branches were recognized. The Whole trunk was exposed, from the styloid foramen till its divisions; the length was determined with a sterile gauge (thread), then measured and recorded with a caliper. The FN branch pattern is photographed and schematic images are drawn (Fig. 2). The parotid gland is pushed forward with a retractor and gently splits from behind; peal the parotid gland from above downward. When the gland was removed, the parotid duct was localized in the mid-point, and then it was divided and ligated (Fig. 3). After identifying the main trunk of the facial nerve, the overlaid parotid tissue is carefully removed from the facial nerve. The nerve branches are dissected sequentially until the entire superficial lobe of the parotid gland, located on the lateral FN side is released. This procedure completes the superficial parotidectomy. If complete parotidectomy is indicated, the procedure is augmented by careful separation of the main FN trunk and branches from underlying parotid tissue. This allows salivary tissue to be delivered with preservation of the facial nerve and its function.

After parotidectomy and homeostasis, the skin wound closed in two layers. Redivak (negative-suction) drain has been used in all cases. When the drain volume was less than 25 ml per day, the drain was removed and the patient was discharged the next day. The skin subcuticular stitch was removed in the 7th postoperative day. Follow-up was recommended to all patients.

**Fig. 4:**
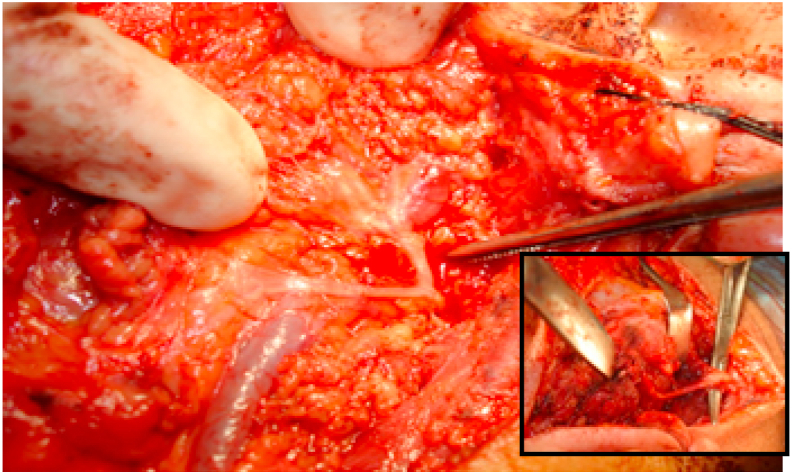
Type I: single FNT that divides into two main divisions, temporofacial and cervicofacial which later divides into branches.

**Fig. 5:**
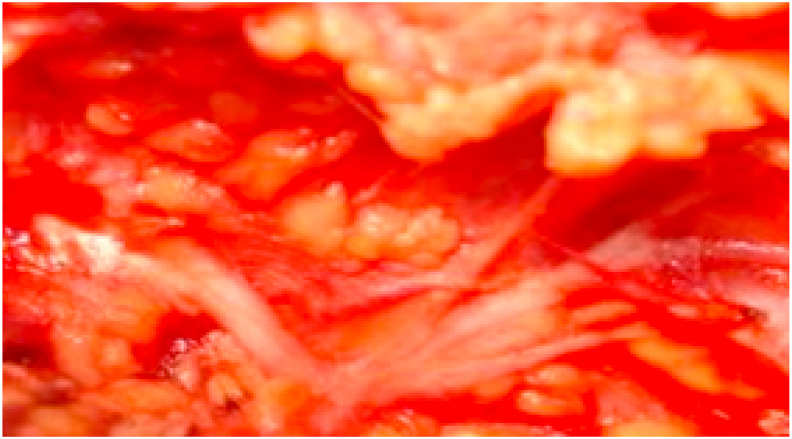
Type II: single FNT that divides directly into final branches without the tow main divisions.

**Fig. 6:**
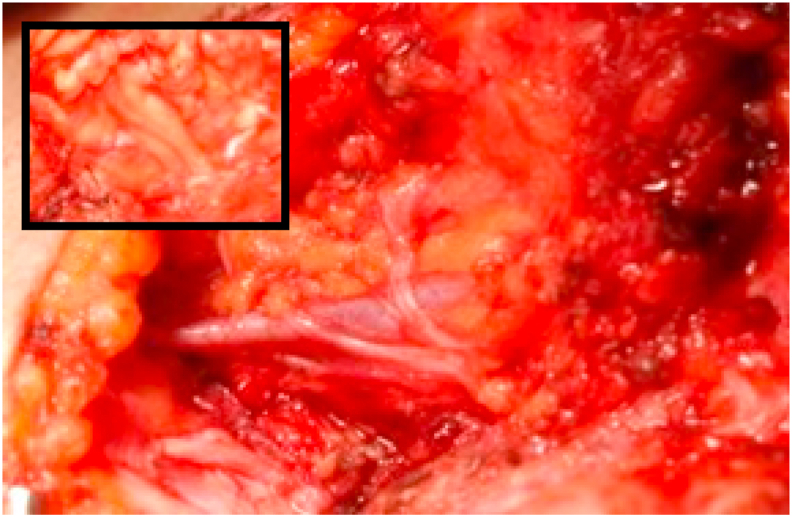
Type III: separate double trunks of FN each give final branches.

**Fig. 7:**
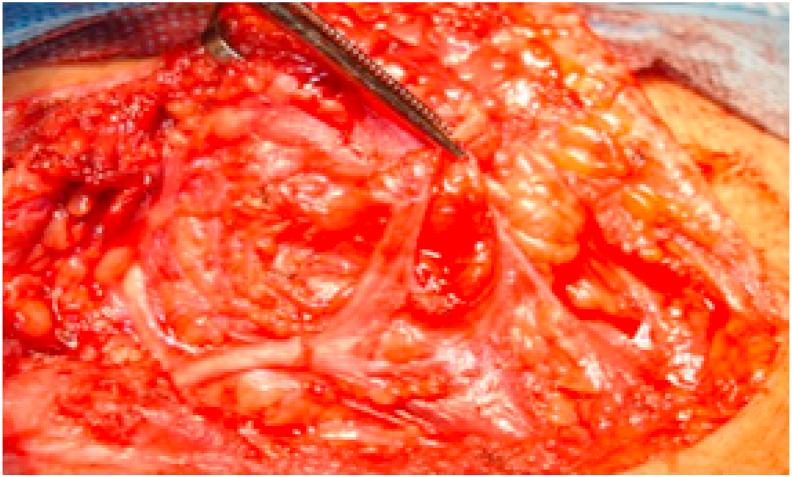
Loop communication between buccal and mandibular branches.

In this study we firstly inspect FN trunk anatomy; with regards to its length, structure of the fractions (single or separated double trunks, or divided directly into branches) (Fig. 4, Fig. 5, Fig. 6). Secondly, we determine the exact location of the FNT with descriptive landmarks: TP and TMP (Fig. 1). Third investigation; we examine whether there is a connection between the branches (Fig. 7).

According to our findings, depending on the anatomical variations in the FTN branching pattern, we assumed a new classification (Table 1); type I: single FNT with 2 main divisions; cervicofacial and temporofacial, which later subdivided into further branches (Fig. 4), type II: single FNT that divides directly into terminal branches without the two main divisions (Fig. 5), type III: separate double trunks of FN each gives final branches (Fig. 6). Each type subdivided into; A: when the FNT length 1–10 mm, B: FNT length 11–20 mm, C: FNT length 21–30 mm. Then each type was further subdivided into 1: when there is no communication between the branches, 2: when there is communication between the branches (Fig. 2).

**Table 1:** age and gender distribution of patients.

Variable	No.	%
Age (year)		
1–10	10	2.2
11–20	40	8.7
21–30	100	21.6
31–40	70	15.3
41–50	70	15.3
51–60	110	23.9
61–70	50	10.8
71–80	10	2.2
Total	460	100.0
**Gender**		
Male	270	58.7
Female	190	41.3
Total	460	100.0

The intraoperative complications like transection of facial nerve or rupture capsule of parotid tumor or incomplete resection of parotid tumor were not reported.

Patients were followed up including outpatient visit after 1 moth, 3 months, and then every year, for assessing the early postoperative complications such as facial nerve palsy, hemorrhage, infection, trismus, parotid fistula, and late complication like recurrence of the tumour.

The data of patients were saved in Excel Software program. The results were organized in an appropriate table and figures in numbers and percentages and reviewed by statistician.

## 2 Results

This study included 460 Iraqi patients with parotid tumors with mean age of (49.2 years) and range of 1–80 years; age groups distribution revealed that 2.2% of patients at the age 1–11 years, 8.7% at 11–20 years, 21.6% at age of 21–30 years, and almost two thirds (65.3%) of the patients aged more than 30 years. Moreover, males were dominant with a male to female ratio of 1.4 to one (Table 1).

The side of parotid tumour in studied patients was right in 58.7% of them and left in 41.3% of them. Facial nerve classification types among Iraqi patients with parotid tumors were type I (78.2%), type II (15.2%) and type III (6.6%) (Table 2).

**Table 2:** Facial nerve trunk characteristics of patients.

Variable	No.	%
Side		
Right	270	58.7
Left	190	41.3
Total	460	100.0
**Types**		
Type I	360	78.2
Type II	70	15.2
Type III	30	6.6
Total	460	100.0

Regarding characteristics of facial nerve trunk classifications; length of FNT was 0.1–1 cm in 54.3% of patients, 1.1–2 cm in 41.3% of patients and 2.1–3 cm in 4.4% of patients. The site of FNT was the midpoint between the TP and TMP in 64.3% of patients and in 20.4% was 2 mm away from the midpoint towards the tip of mastoid process, while in 15.3% was 2 mm away from the midpoint towards the TP. No communications between FNT branches were observed in 84.7% of patients, while the communications were observed among 15.3% of them (Table 3).

**Table 3:** Facial nerve trunk types characteristics of patients.

Variable	No.	%
Length		
0.1–1 cm	250	54.3
1.1–2 cm	190	41.3
2.1–3 cm	20	4.4
Total	460	100.0
**Site**		
Mid	296	64.3
Below	94	20.4
Above	70	15.3
Total	460	100.0
**Communication**		
Yes	70	15.3
No	390	84.7
Total	460	100.0

After assigning each facial nerve according to our classification, the results were as follows: Type IA1 160(34.78%); 90(19.56%) males, 70(15.22%) females. IA2 20(4.35%), all were males and the communication was always a loop between mandibular and buccal branches of FN. Type IB1 150(32.61%); 90(19.56%) males, 60(13.04%) females. IB2 10(2.17%), all were females and the communication was always a loop between the upper and lower divisions of FNT. IC1 20(4.35%), all were males. There were no cases of type IC2. Type IIA1 10(2.17%), all were males. IIA2 30(6.52%); 20(4.35%) males, 10(2.17%) females, and the communication was always a loop between the buccal and mandibular branch. IIB1 30(6.52%); 10(2.17%) males, 20(4.35%) females. There were no cases of type IIB2, IIC1, and IIC2. Type IIIA1 20(4.35%); 10(2.17%) males, 10(2.17%) females. IIIA2 10(2.17%) all were females and the communication was always a loop between the upper and lower divisions of FNT. There was no cases of type IIIB1, IIIB2, IIIC1, and IIIC2 (Table 1) (Fig. 2). So in total type I was 360(78.26%), type II was 70(15.22%), and type III was 30(6.52%). Our results of facial nerve branching pattern in accordance with classification of Davis et al. (7), was type I the first most common branching pattern 390(84.78%). While the second most common branching pattern in this study was the presence of anastomotic connection between the mandibular and buccal branches of FN in 50 cases (10.87%), and this branching pattern was not reported by others. The third most common branching pattern was type III, contributed for 20 (4.34%), In this study, neither type II, type IV, type V, nor type VI had been reported, (Table 4).

**Table 4:** Facial nerve branching pattern types.

Variables		Type I				Type II				Type III			
		1		2		1		2		1		2	
		No.	%	No.	%	No.	%	No.	%	No.	%	No.	%
A/FNT length (1–10 mm)	Male	90	19.6	20	4.35	10	2.17	20	4.35	10	2.17	0	0
	Female	70	15.2	0	0	0	0	10	2.17	10	2.17	10	2.17
	Total	160	34.8	20	4.35	10	2.17	30	6.52	20	4.35	10	2.17
B/FNT length (11–20 mm)	Male	90	19.6	0	0	10	2.17	0	0	0	0	0	0
	Female	60	13.0	10	2.17	20	4.35	0	0	0	0	0	0
	Total	150	32.6	10	2.17	30	6.52	0	0	0	0	0	0
C/FNT length (21–30 mm)	Male	20	4.4	0	0	0	0	0	0	0	0	0	0
	Female	0	0.0	0	0	0	0	0	0	0	0	0	0
	Total	20	4.4	0	0	0	0	0	0	0	0	0	0


Type I: single FNT that divides into two main divisions, Type II: single FNT that divides directly into final branches, Type III separate double trunks of FN. 1: no communication between the branches, 2: there is communication between the branches.

The postoperative complications were recorded only for 17 (3.7%) of patients with parotid tumors; transient facial nerve palsy was the common complication (41.1%) followed by hypoesthesia of greater auricular nerve (29.4%) and parotid fistula (11.8%), etc.(Table 5)

**Table 5:** Postoperative complications.

Variable	No.	%
Transient facial nerve palsy	7	41.1
Hypoesthesia of greater auricular nerve	5	29.4
Frey's syndrome	1	5.9
Parotid fistula	2	11.8
Seroma	1	5.9
Recurrent tumour	1	5.9
Total no. of patients	17	100.0

## 3 Discussion

Facial nerve palsy after parotidectomy is still a common complication. Recognition of FNT is important to maintain its function, which is a challenge for the surgeon due to unpredictable changes in the pattern of facial nerve branching [3,4].

Anatomy of FN was investigated previously in many studies [8,[13], [14], [15]] and different landmarks were studied by surgical and anatomical studies, for assistance of surgeons to safely recognize the FN, for instance, some authors stated that posterior belly of digastric muscle (DGM) is good reference landmark for recognition of FNT [16], while other investigators reported that styloid base and the posterior belly of DGM origin considered better and safe bone landmarks for the identification of FNT [17], as a consistent superficial bony landmarks, the mandible angle and the tip of mastoid process were considered by other studies to identify the trunk [18]. However, there is still much debate about the most reliable and safest landmarks. Authors, proposed that the best landmark should be easily palpated and superficial in location which not need complete deep tissue resections [18,19].

Bony landmarks are the most reliable anatomical guides owing to their rigid and consistent location. In this study the TP and the tip of mastoid process have been chosen as they met all the criteria, in most of patients (64.35%) the FNT was in the midpoint between the TP and TMP, in 20.43% was 2 mm away from the midpoint towards the TMP, and in 15.22% was 2 mm away from the midpoint towards the TP. So if we didn't identify the FNT in the midpoint, we go first 2 mm below, if not identified we go 2 mm above the midpoint. Other researchers, Stankevicius et al. and Pather et al., depend on measuring the distance between the FNT and angle of mandible, the mean distance was 36.45 ± 4.14 mm, and 38.10 ± 3.10 mm respectively [18,19]. While Stankevicius et al. and Farahvash et al., measured the distance of FNT – TMP was found to be 12.52 ± 2.30 mm, and 11.81 ± 2.01 mm respectively [18,20], a mean of 9.30 ± 0.9 mm as reported for the FNT-TP-distance [18]. We didn't depend on this way of measurements because we think it's not practical for the surgeon to make this measurement in live patients as in cadavers. Also in this study we concentrated on describing the variations in the anatomy of the FNT, including its length and its fraction pattern, as this is the most important step in doing parotidectomy. We found three types of FNT morphology. type I which is described by all researchers, it is the most common type were there was single trunk of facial nerve present, in some studies this represent 91.4% of the cases [8], Our results were much different, and this type was present in 78.26% of cases. Type II was persent in 15.22%, and was reported by one researcher only, he found in foetal specimens 12% of cases the facial nerve directly gives 5 terminal branches [21]. While it was described that FNT split as trifurcation in 9% [18] and Myint reported 3.8% trifurcation only [22]. Type III where there is separate double trunks of FN, constitutes 6.52% of our cases, while this type recognized by other researchers to constitute 8.57%,9%, 12% of the cases respectively [8,18,23].

Botman and Jongkees concluded that within the mastoid segment, FN could be splitted giving 2 or 3 FNTs that exit separately, through osseous foramen [24]. These types are operatively of considerable importance. Therefore, surgeons should always be aware of the potential of types II and III of FNT and take precautions to avoid injuring them.

In this study, length of 1–10 mm of main FNT was the commonest reported length, secondly in frequency was 11–20 mm, and the least common was 21–30 mm. While in other studies the length of 16–20 mm was the most common, followed by 11–15 mm and the least common was more than 20 mm, while none had the main trunk less than 10 mm [11,15,25]. Finally we studied the branching pattern and if there are communications between them, different studies and literatures assessed and studied this subject, nonetheless, majority of these studies, compared the branching pattern according to Davis classification [7], which classified the branching pattern into 6 different types. The most common pattern in our study according to Davis classification was type I (84.78%), and this is comparable to other studies [8,26,27], while lower incidence of type I was reported by Davis et al. and Myint et al. [7,22].

Furthermore, the second more frequent type was Type III branching-pattern according to Khaliq et al., and the first most common pattern according to Davis and Myint et al., respectively [7,8,22]. In our study this branching pattern was the third most common. While the second most common branching pattern in this study was the presence of anastomotic connection between the buccal and mandibular FN branches which contributed for (10.87%), and this branching pattern was not reported by others. Type II, IV, V, and VI were not reported in this study. However, other studies documented that types IV, V and VI were the least frequent types of branching patterns, despite, higher rate of type VI branching patterns was documented by a study conducted by Myint et al. [22].

The main limitations in this study were the design of study as cross sectional with lack of temporality in associations, loss to follow up of patients and reporting of postoperative complications and generalizability of findings as our study were done in the hospitals of Baghdad (Capital) and not in all Iraqi cities. However the sample was collected from different surgical centers and could be representative.

In conclusion, a profound variations had been observed among Iraqis in the pattern of facial nerve branching that have not been previously reported. It can be clearly seen that the results differ in different studies. The topography of the facial nerve during parotidectomy has always been a problem for surgeons due to unknown and unexpected differences in the patterns of the facial nerve branches. The main recommendation of present study was the familiarity with these common differences in facial anatomy provides the surgeon with useful information about accurate dissections, facial nerve preservation, and complete removal of the parotid gland neoplasm.

## Funding

Financial resources of researcher.

## Please state any conflicts of interest

None.

## Please state any sources of funding for your research

I declared all sources of funding. and declare the role of study sponsors, if any, in the collection, analysis and interpretation of data; in the writing of the manuscript; and in the decision to submit the manuscript for publication.

## Ethical Approval

- Informed written consent was obtained after explaining the nature of the operation and its risks.

- The approval of ethics committee was obtained from Health Ethics Committee in Baghdad Medical city teaching hospital.

- Ethical considerations were obtained according to Helsinki Declaration.

## Consent

Written informed consent was obtained from the patient for publication.

Informed consent paper:

Dear patients:

This study aimed to assess facial nerve branching variation in Iraqi population.

This study obtained information directly from you and other data during the surgical operation.

Your participation in study as volunteer and not mandatory or financed.

You have the right to not participate in this study and this will not affect the quality of the surgical operation.

You have the right to withdrawal from the study at any time of research.

The researcher respects your confidentiality.

Researcher.

## Author contribution

Please specify the contribution of each author to the paper, e.g. study concept or design, data collection, data analysis or interpretation, writing the paper, others, who have contributed in other ways should be listed as contributors.

Single author, did the study concept or design, data collection, data analysis or interpretation, writing the paper.

## Registration of Research Studies

1 Name of the registry:

www.researchregistry.com.

2 Unique Identifying number or registration ID:

6255.

3 Hyperlink to registration:

https://www.researchregistry.com/browse-the-registry#home.

( Researchregistry 6255. Available at: www.researchregistry.com

https://www.researchregistry.com/browse-the-registry#home)

## Guarantor

No Guarantor.

## Declaration of competing interest

Declared none.
