# Supplementary material for: New classification of branching pattern of facial nerve during parotidectomy: A cross sectional study
Source: Ann Med Surg (Lond). 2021 Jan 14;62:190–6. doi: 10.1016/j.amsu.2021.01.006 (PMC7843363; doi:10.1016/j.amsu.2021.01.006)
Supplement: Multimedia component 1 [file mmc1.doc]

**STROCSS Checklist**

| **Outline** | **Page number** |
| --- | --- |
| 1. [Highlights](https://www.sciencedirect.com/science/article/pii/S1743919117312463?via%3Dihub#abs0015) | Sent as separate folder |
| **2. Abstract** | (1-2) |
| **3.** [Keywords](https://www.sciencedirect.com/science/article/pii/S1743919117312463?via%3Dihub#kwrds0010) | (2) |
| **4. Introduction** | (2-3-4) |
| **5.  Methods** | (4-5-6-7) |
| **6. Participant selection** | (4) |
| **7. Results** | (7-8-9-10-11) |
| **8. Conclusion** | (2, 14) |
| **9.** [Ethical approval](https://www.sciencedirect.com/science/article/pii/S1743919117312463?via%3Dihub#sec6) | (4) |
| **10.** [Funding](https://www.sciencedirect.com/science/article/pii/S1743919117312463?via%3Dihub#sec7) | (15) |
| **11.** [Conflicts of interest](https://www.sciencedirect.com/science/article/pii/S1743919117312463?via%3Dihub#sec9) | (15) |
| **12.** [Research registration unique identifying number](https://www.sciencedirect.com/science/article/pii/S1743919117312463?via%3Dihub#sec10) | (4)  **-** Reference number [12], page (16) |
| **13.** [Guarantor](https://www.sciencedirect.com/science/article/pii/S1743919117312463?via%3Dihub#sec11) | (15) |
| **14.** [STROCSS group participants](https://www.sciencedirect.com/science/article/pii/S1743919117312463?via%3Dihub#appsec1) | (4)  **-** Reference number [11], page (16) |
| **15.** [References](https://www.sciencedirect.com/science/article/pii/S1743919117312463?via%3Dihub#cebib0010) | (15-18) |
